# Hebbian-inspired rewiring of a random network replicates pattern of selectivity seen in PFC

**DOI:** 10.1186/1471-2202-15-S1-P160

**Published:** 2014-07-21

**Authors:** Grace Lindsay, Mattia Rigotti, Melissa R Warden, Earl K Miller, Stefano Fusi

**Affiliations:** 1 Department of Neuroscience, Columbia University, New York, NY 10026, USA; 2 Department of Bioengineering, Stanford University, Stanford, CA 94305, USA; 3 The Picower Institute for Learning and Memory & Department of Brain and Cognitive Sciences, Massachusetts Institute of Technology, Cambridge, MA 02139, USA

## 

Responses of neurons in pre-frontal cortex (PFC) are very diverse and often depend on complex non-linear combinations of task-relevant variables (a property known as mixed selectivity). We recently showed that this type of selectivity is a signature of high dimensional neural representations and can be important for performing complex cognitive tasks [1]. Previous modeling work has shown that mixed selectivity can arise when cells receive fixed random synaptic inputs from populations that represent the task-relevant variables (see, e.g. [2]). Here we show by analyzing the data of [3] that these simple models only partially explain the mixed selectivity observed in the data.

The data we analyzed is from a delayed-response task wherein monkeys are presented a sequence of two image cues on each trial, followed by a 1-second delay period after which the monkey either saccades to them in the correct order when a display of images is presented (“recall task”) or reports if a presented sequence is a match (“recognition task”). We characterized the statistics of the recorded PFC responses by determining the number of mixed selective cells. Specifically, we performed a 3-way ANOVA test on firing rate responses using the three task parameters (task type, cue 1, and cue 2) as factors. The requirement for mixed selectivity was for at least one interaction term to have a significant coefficient (p-value <0.05). Cells were classified as “pure selectivity” neurons if they had at least one significant pure task parameter term and no significant interaction term.

Initially, we modeled PFC as a population of cells receiving random connections from a population of binary input cells each representing a task-relevant variable: a task type, cue 1 image, or cue 2 image. However the statistics of the neuronal responses of the model only partially reproduced the data.

**Figure 1:**
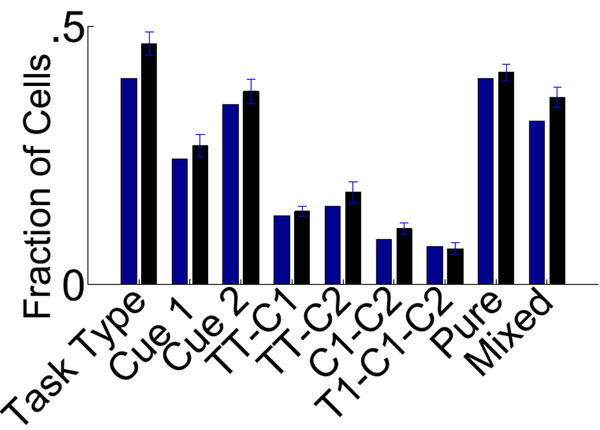
Result of ANOVA analysis: For each term in the ANOVA, fraction of cells whose best fit results in a significant coefficient for that term (blue: data, black: best-fit model. On the left: the 7 ANOVA terms,on the right: percentage of cells that fall into the two categories as described above).

For this reason we introduced structure to the randomly-connected network in the form of rewiring, which creates specialization in the inputs to individual cells. The rewiring procedure works by identifying which task-relevant variable best characterizes the response of a given PFC neuron. Connections are then redirected to further increase the input from the population representing this variable (and consequently decrease other inputs). The extent of this rewiring is determined by a single parameter: the probability of redirecting a connection. The rewiring can be interpreted as the result of competitive Hebbian learning. By varying the probability of rewiring, this initially-unstructured model can be tuned to accurately reproduce the pattern of selectivity seen in the data (Figure 1), by increasing mixed selectivity and decreasing pure selectivity. Our analysis suggests that random connectivity is an important substrate for generating diverse responses. However, neurons also exhibit some degree of specialization, which is likely to be the result of plasticity. We hypothesize this specialization is important to increase robustness to noise and should reflect behavioral adaptation to the task. Accordingly, we predict that experiments investigating the evolution of the neural population response throughout behavioral training should reveal a correlation between task performance and our rewiring parameter.
